# Supplementary figures and images for: Non-muscle myosins control radial glial basal endfeet to mediate interneuron organization
Source: PLoS Biol. 2023 Feb 28;21(2):e3001926. doi: 10.1371/journal.pbio.3001926 (PMC9974137; doi:10.1371/journal.pbio.3001926)

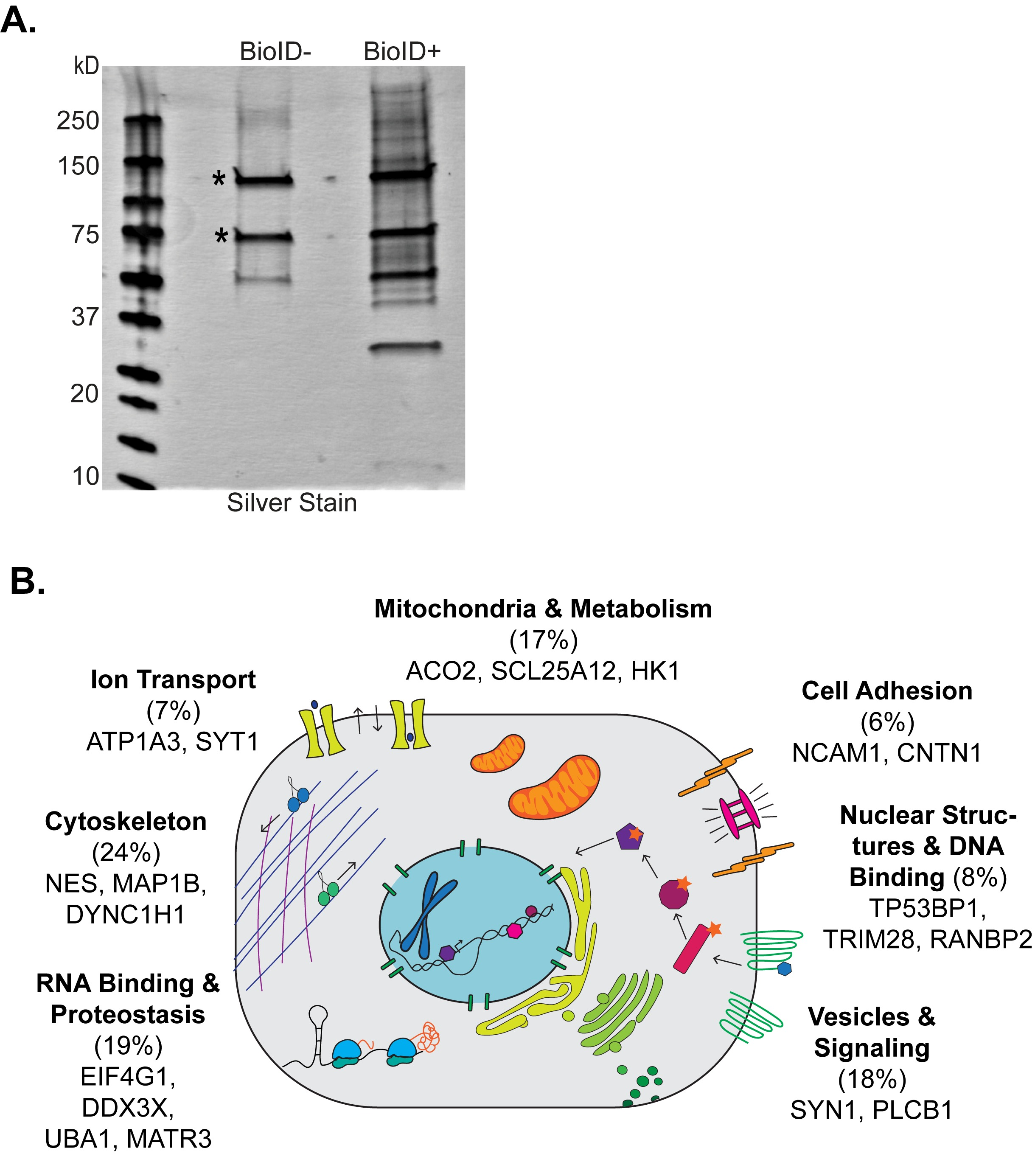

Supplement: S1 Fig — (A) Gradient 4% to 20% Silver stain gel of E15.5 whole RGC BioID− and BioID+ samples following affinity purification shows more biotinylated proteins in the BioID+ condition. Pool of 8 cortices per condition. Asterisks denote endogenously biotinylated carboxylases. (B) Cartoon representation of the diverse categories of proteins labeled by cytoplasmic, untethered BirA*. (TIF) [file pbio.3001926.s001.tif]

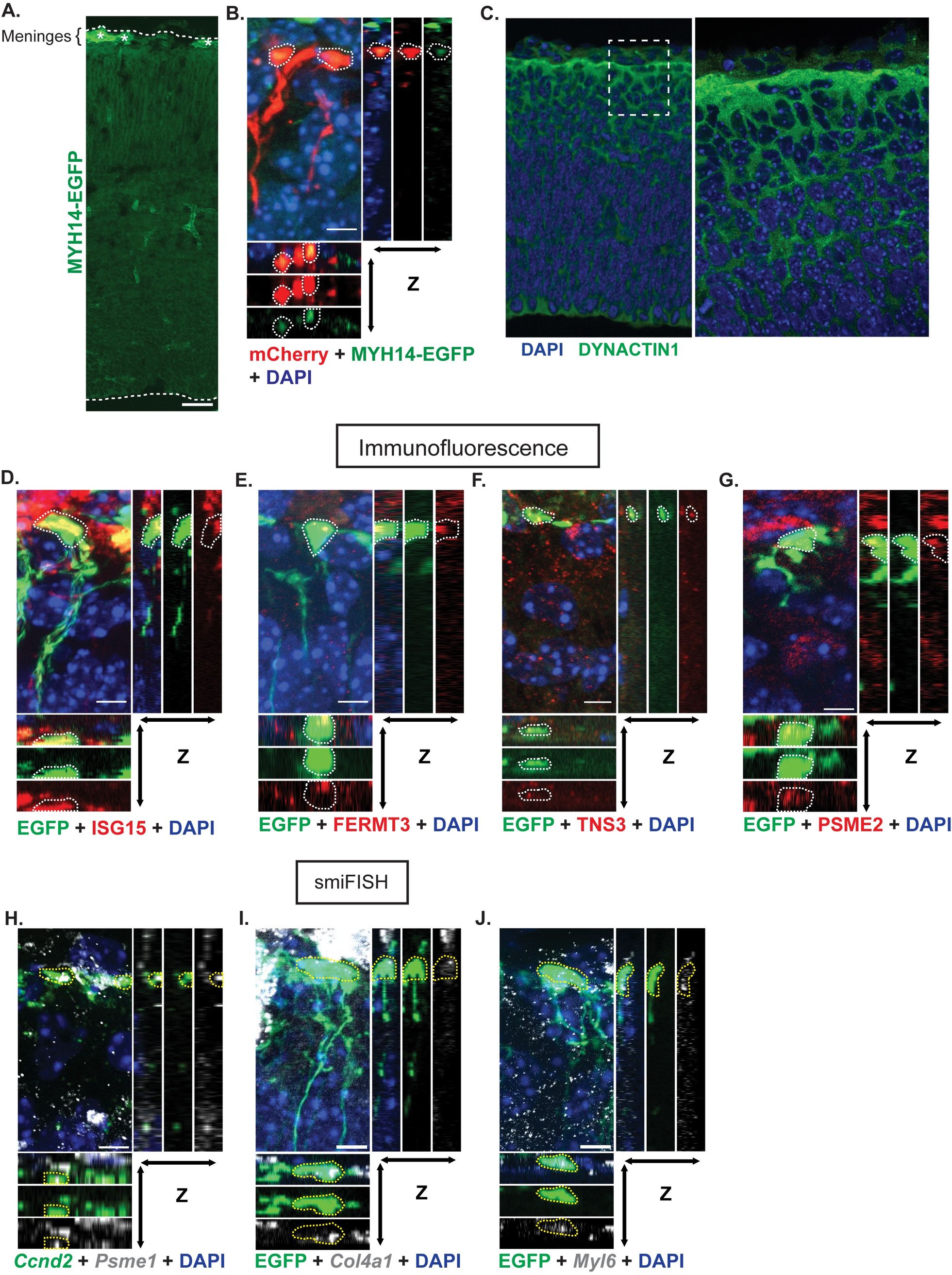

Supplement: S2 Fig — (A) Cortical column showing MYH14 (green) expression at E15.5 marked by MYH14-EGFP mouse. Dotted lines represent pial and ventricular borders. Asterisks denote background signal from the meninges. (B) Colocalization of MYH14-EGFP with mCherry-labeled endfeet (red) shown by orthogonal views. (C) Anti-dynactin1 labeling throughout the cortex and at the pia. Dotted box outlines region of interest for zoom (Right). (D-G) Protein (red) colocalization with EGFP-labeled endfeet (green) shown by orthogonal views. Endfeet outlined by white dashed lines. ISG15 (D), FERMT3 (E), TNS3 (F), PSME2 (G). colocalization with EGFP-labeled endfeet (green) shown by orthogonal views. (H) Psme1 smiFISH (grey) colocalization with Ccnd2 smiFISH (green) labeling endfeet shown by orthogonal views. Endfeet outlined by white dashed lines. (I, J) mRNA (grey) colocalization with EGFP labeled endfeet (green) by orthogonal views. Endfeet outlined by white dashed lines. Col4a1 (I) and Myl6 (J). n = 2 to 3 brains, 3 sections per brain (A-J). Scale bar: (A) 50 μm; (B) 10 μm; (D-J) 5 μm. (TIF) [file pbio.3001926.s002.tif]

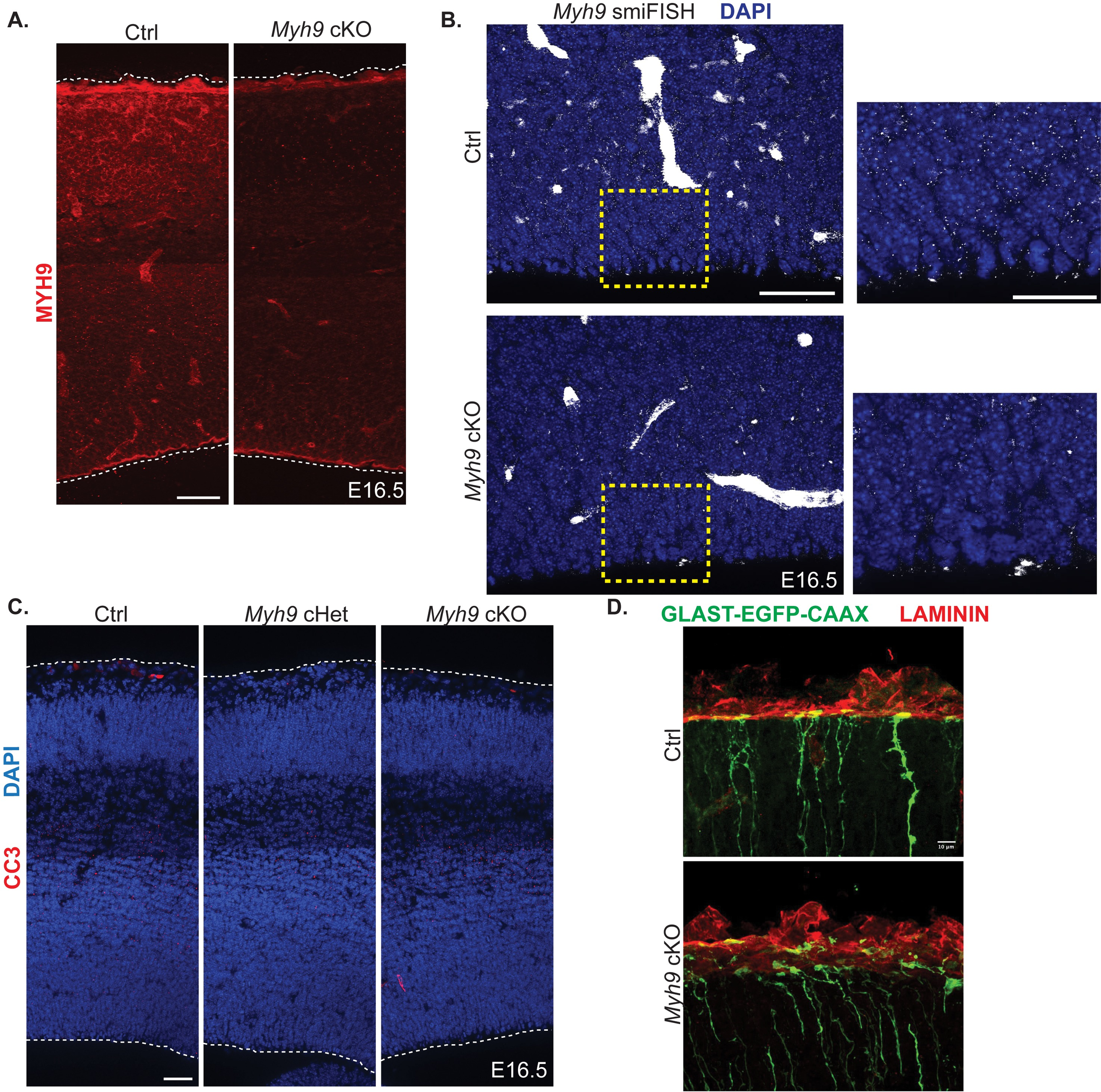

Supplement: S3 Fig — (A) MYH9 expression (red) in Ctrl and Myh9 cKO cortical columns at E16.5. Dotted lines represent pial and ventricular borders. n = 3 per genotype from 2 litters. (B) Myh9 expression by smiFISH (grey) in Ctrl and cKO brains at E16.5. Yellow dashed line represents area of interest expanded (Right). n = 3 per genotype from 2 litters. (C) CC3 (red) and DAPI (blue) staining of Ctrl, Myh9 cHet, and Myh9 cKO cortical columns at E16.5. Dotted lines represent pial and ventricular borders. n = 3 per genotype from 2 litters. (D) Representative images of Ctrl and Myh9 cKO endfeet (labeled by GLAST-EGFP-CAAX in green) relative to the BM (labeled by laminin in red). n = 5 Ctrl from 3 litters and 3 cKO from 2 litters. Scale bar: (A, C, B-left) 50 μm; (B-right) 25 μm; (D) 10 μm. (TIF) [file pbio.3001926.s003.tif]

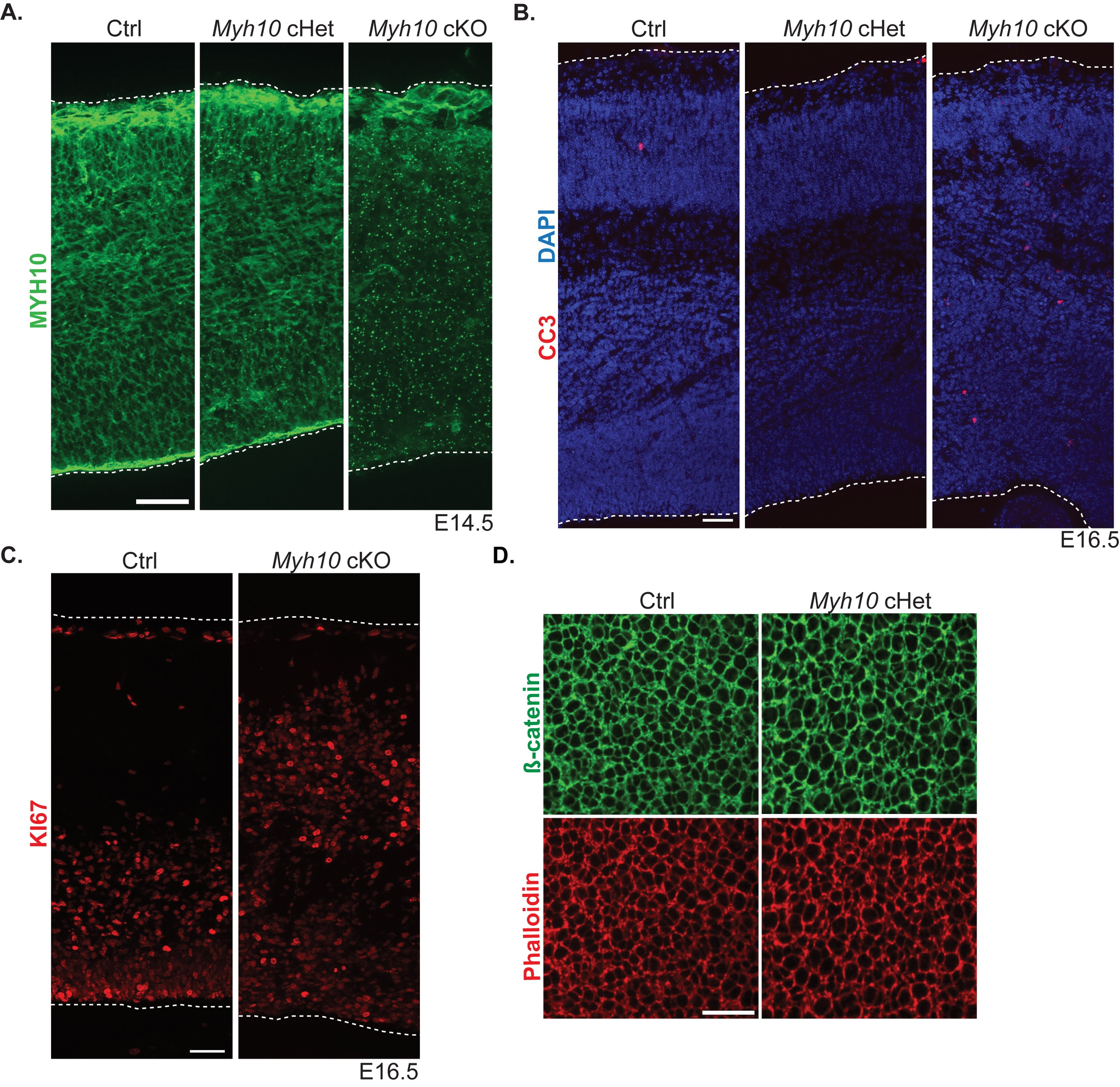

Supplement: S4 Fig — (A) MYH10 expression (green) in Ctrl, Myh10 cHet, and Myh10 cKO cortical columns at E14.5. n = 3 per genotype from 1 litter. (B) CC3 (red) staining of Ctrl, Myh10 cHet, and Myh10 cKO cortical columns at E16.5. n = 5 per genotype from 3 litters. (C) KI67 staining (red) of Ctrl and Myh10 cKO brains at E16.5. n = 4 Ctrl, 3 cKO from 2 litters. (D) Comparison of Ctrl and cHet en face apical endfeet labeled with β-catenin (green) and Phalloidin (red). Note: Ctrl images are the same as those from Fig 5L. n = 9 Ctrl from 3 litters and 3 cHet from 2 litters. Dotted lines represent pial and ventricular borders. (A, B, C) Three sections imaged per brain (A, B, C). Scale bar: (A, B, C) 50 μm; (D) 25 μm. (TIF) [file pbio.3001926.s004.tif]

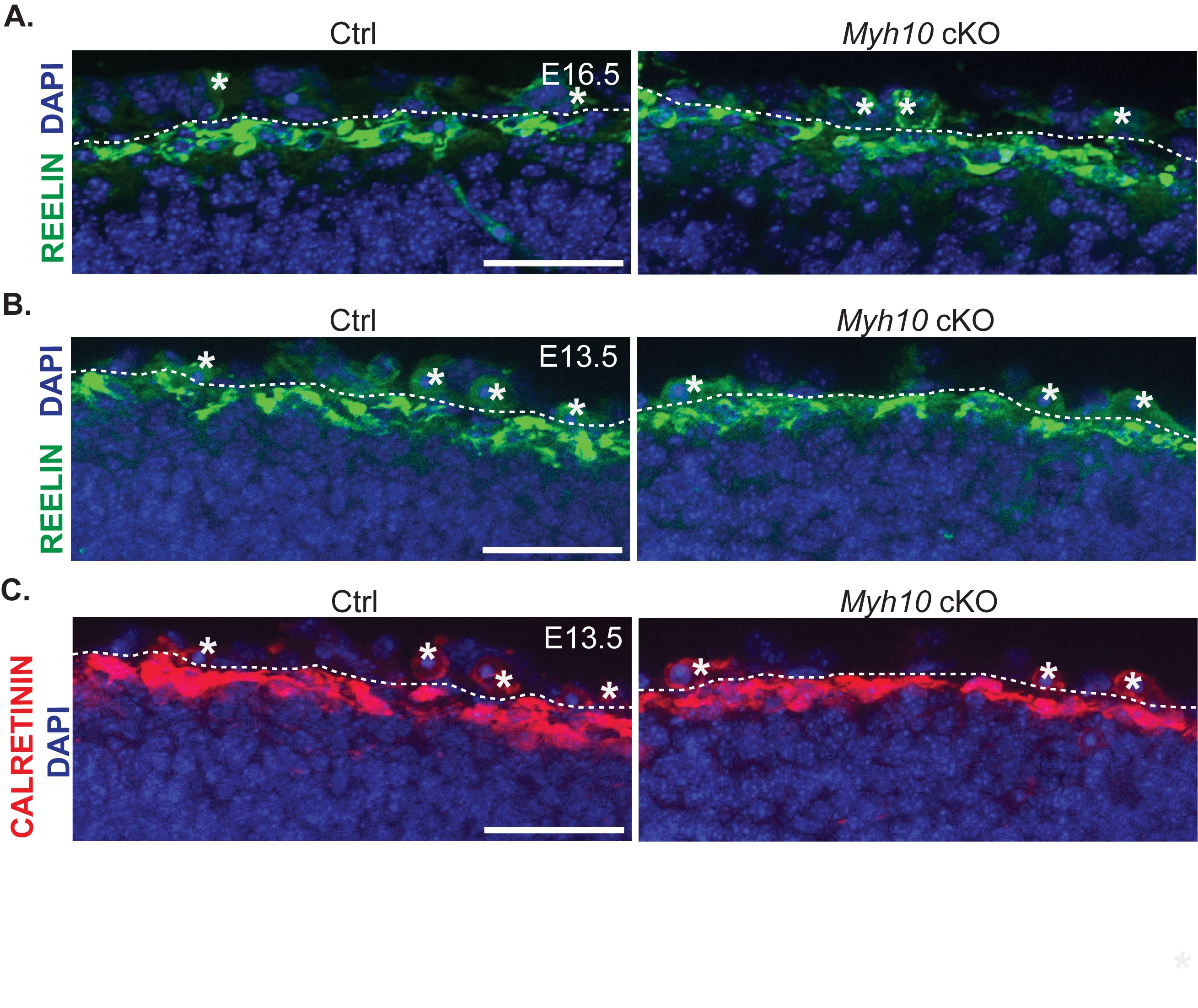

Supplement: S5 Fig — (A) Reelin expression (green) in the MZ of Ctrl and Myh10 cKO brains at E16.5. n = 6 per genotype from 2 litters. (B) Reelin expression (green) in the MZ of Ctrl and Myh10 cKO brains at E13.5. n = 3 per genotype from 1 litter. (C) Calretinin expression (red) in the MZ of Ctrl and Myh10 cKO brains at E13.5. n = 3 per genotype from 1 litter. Scale bar: (A, B, C) 50 μm. Dotted lines represent border between pia and MZ (A, B, C). (TIF) [file pbio.3001926.s005.tif]
