# Supplementary material for: Non-muscle myosins control radial glial basal endfeet to mediate interneuron organization
Source: PLoS Biol. 2023 Feb 28;21(2):e3001926. doi: 10.1371/journal.pbio.3001926 (PMC9974137; doi:10.1371/journal.pbio.3001926)
Supplement: S1 Raw Images — (PDF) [file pbio.3001926.s019.pdf]

Gel For S1A Fig

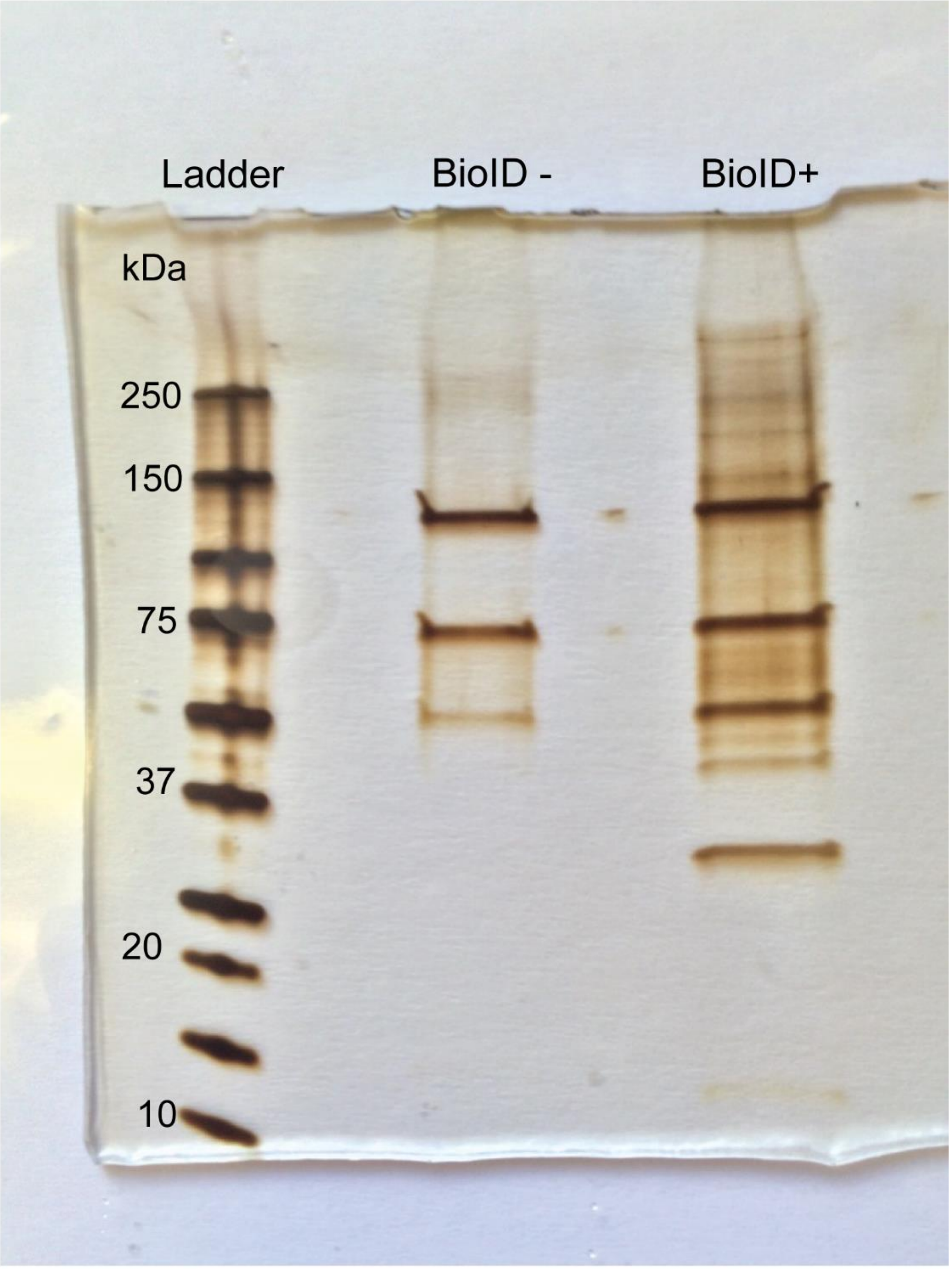

Image was captured with a cell phone camera

Streptavadin-HRP Western Blot used for Fig 1F

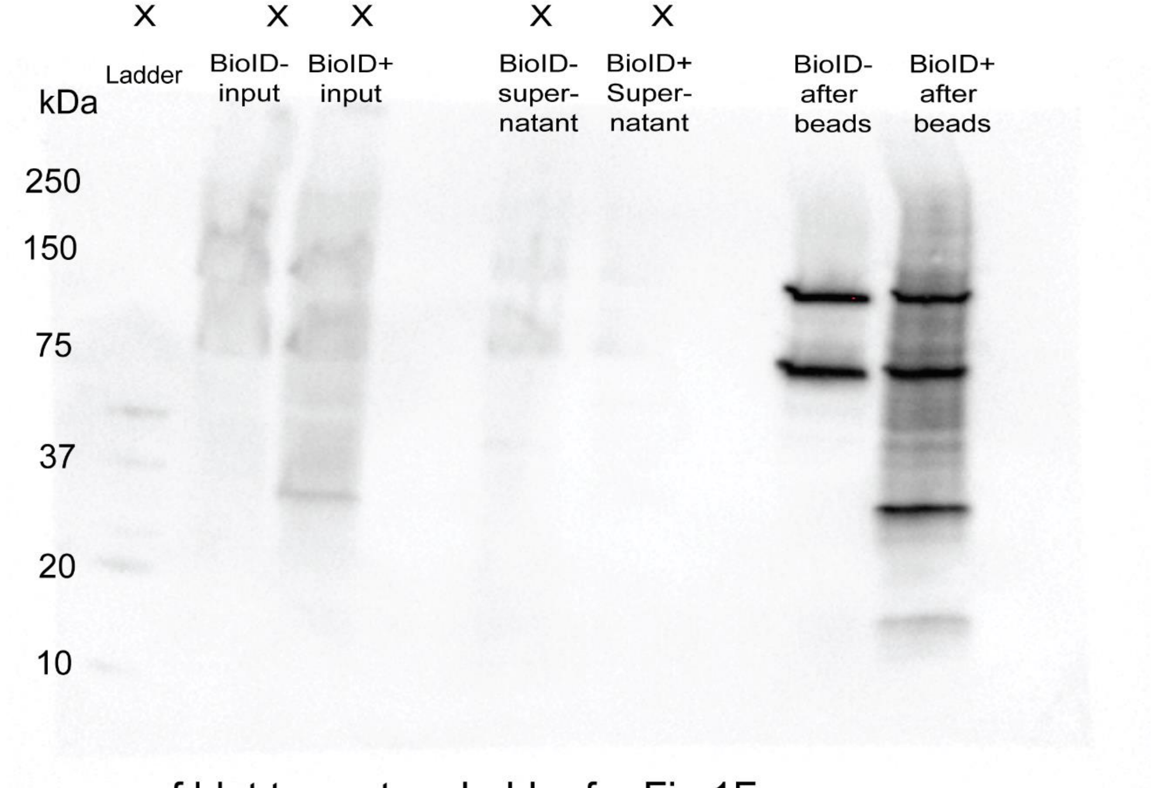

UV exposure of blot to capture ladder for Fig 1F

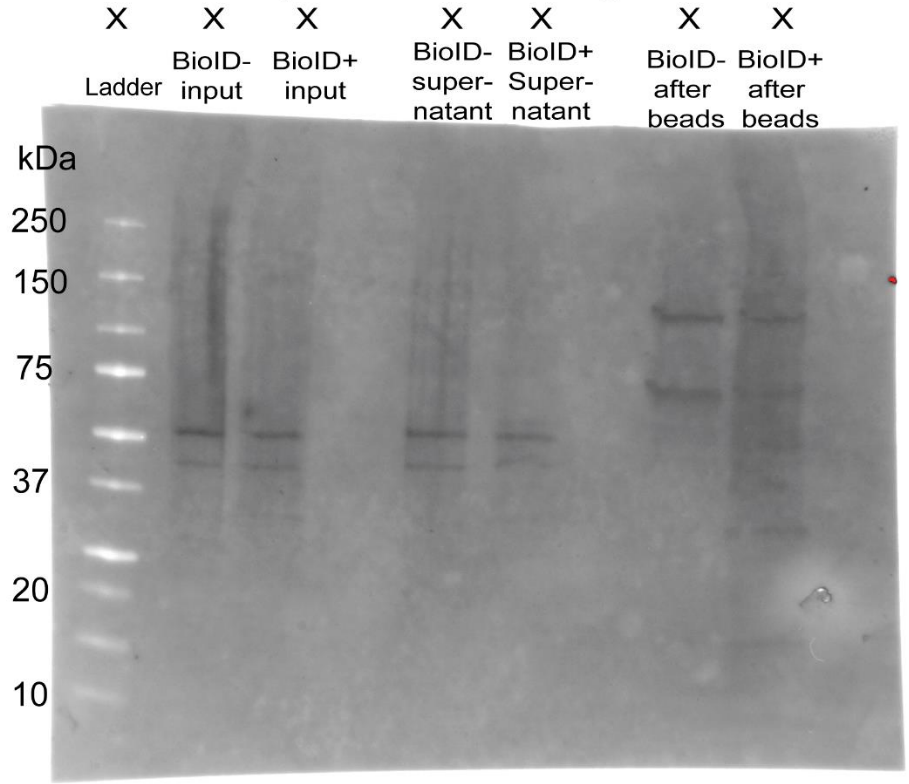

Both images captured with BioRad ChemiDoc XRS+ imager
