# Supplementary material for: Non-muscle myosins control radial glial basal endfeet to mediate interneuron organization
Source: PLoS Biol. 2023 Feb 28;21(2):e3001926. doi: 10.1371/journal.pbio.3001926 (PMC9974137; doi:10.1371/journal.pbio.3001926)
Supplement: Attachment — Submitted filename: Response to Reviewers.pdf [file pbio.3001926.s020.pdf]

Reviewer #1: In this study, D'Arcy et al. set out to define the local proteome of radial glial cell (RGC) endfeet in the developing mouse neocortex. To this end, they used a creative approach combining RGC endfoot microdissection, a method which allows for mechanical separation of the basal endfoot from the rest of the RGC, with BioID proximity-labeling proteomics. In doing so, they identified a cohort of proteins, including components of extracellular matrix, ubiquitin-proteasome pathway, and cytoskeletal regulators, that are enriched in RGC basal endfeet. Among them, the non-muscle myosin heavy chain II (NMHC II) isoforms, namely NMHC IIA (MYH9) and NMHC IIB (MYH10), were among the most abundant. This prompted the authors to further characterize the function of these proteins in RGC endfeet, taking advantage of Myh9lox/lox;Emx1-Cre and Myh10lox/lox;Emx1-Cre mutant mice. By performing loss of function studies, they found that Myh9 is important for the regulation of RGC complexity and endfoot organization relative to the basement membrane (BM) while Myh10 is required for maintaining RGC basal and apical endfeet attachments. Finally, the authors present data supporting that Myh9 non-cell autonomously regulates interneuron position in the marginal zone (MZ) while Myh10 regulates both interneuron position and number.

This is an interesting study, providing novel insight into the subcellular proteome of RGC basal endfeet. Furthermore, the study sheds novel light on the functions of Myh9 and Myh10 in the regulation of RGC endfoot morphology. The manuscript is well written, and overall the experiments are well executed. There are, however, a number of issues that need to be addressed (see below).

We thank the reviewers for their enthusiasm towards our study and thoughtful suggestions for its improvement.

Specific points:

1) By performing qualitative tandem mass spectrometry on proteins affinity purified from BioID+ and BioID- cortices, the authors found that besides BioID+ specific biotinylated proteins, there were 110 BioID- specific biotinylated proteins (almost 25% of the total biotinylated proteins found in BioID- samples) (Fig S1). Since the BioID- samples are representative of the baseline biotinylation in control mice, one would expect all biotinylated proteins to also appear in the BioID+ samples? What is the identity of these proteins? The authors should comment on this.

We thank the reviewer for this important comment. There can be background associated with the affinity beads. As the reviewer notes, the Venn that was in S1 Fig reports qualitative data, which are based upon just 1 sample each for BioID+ and BioID-. The quantitative analysis reported in our study is based upon 3 BioID+ and 3 BioID- samples (Fig. 1). Indeed, of the 110 proteins specific to the BioID- qualitative sample, only 21 were found in the quantitative dataset. All 21 proteins were present in both the BioID+ and BioID- samples, indicative of background. Because we feel the Venn diagram is confusing and not representing our rigorous quantitative analysis, we have removed it from the manuscript (and instead report the total number of proteins detected). However, if the editors feel it is important, we can include the Venn.

2) The authors show that loss of Myh9 in RGCs leads to decreased branching complexity and protrusion of basal endfeet through the BM. To assess that these defects are not secondary to cell body defects, they examined the effects of Myh9 loss on progenitor cell organization, apoptosis, and neurogenesis at E16.5. While their data support that the basal endfoot phenotypes are not likely the result of defects in these processes, they do not per se exclude a role for Myh9 in any of these processes, as they may be only apparent at later stages of development. The

authors should at least include data examining the impact of Myh9 loss in RGCs on neurogenesis at later stages of development.

We agree that we cannot rule out a possible role for *Myh9* in neurogenesis, evident later in development. We have modified our conclusions that *Myh9* (and *Myh10)* loss does not impact neurogenesis, to specify that this refers only to E16.5, the stage we quantified (lines 308-309, 375, 585-588). Our discussion now includes a paragraph describing the importance of additional characterization of the possible roles of *Myh9* and *Myh10* in neurogenesis (Lines 638-644).

Also, it is not clear whether loss of Myh9 has no effect on the apical endfoot. Magnified images of the apical endfoot and cell body of Myh9 cKO RGCs should be included.

We did not perform *en face* imaging of apical endfeet in *Myh9* cKO cortices (as in *Myh10* cKO), however we do not anticipate a defect in the apical endfoot for two main reasons. First, we do not see basal localization of the RGCs away from the ventricle, as quantified by SOX2+ layer thickness (Fig 4E), suggesting that they are still anchored apically. Additionally, immunofluorescence of coronal sections does not show MYH9 localization at the apical endfeet. In contrast to MYH10, MYH9 signal is not present beyond edge effect (staining artifacts sometimes seen with sections) as demonstrated by signal in the Ctrl and *Myh9* cKO sections (S3 Fig A). We have added a brief discussion of why we don't think *Myh9* impacts apical endfoot (Lines 394-396).

3) The authors present data showing that the basal endfeet of Myh10 cKO RGCs are initially attached at the pia (at E14.5) and then progressively become unattached from the BM (by E15.5). They also found that the apical endfeet of Myh10 cKO RGCs become disorganized and detached from the ventricle (starting at E14.5), leading to more basally located RGCs. Can the authors exclude that the gradual detachment of the basal endfeet is the result of a detachment of the apical endfeet? They should comment on this.

We agree with the reviewer that our data cannot definitively discriminate whether apical endfoot detachment contributes to basal endfoot detachment. We have added this important point to our discussion (Lines 644-648).

Also, previous studies have shown that disorganization/detachment of RGC apical endfeet results in more basally dividing progenitors, ultimately leading to an increase in neurogenesis. The authors report that loss of Myh10 in RGCs does not affect neurogenesis at E16.5. Do they see altered neurogenesis at later developmental time points in the Myh10 cKO animals? If not, how do they explain this?

We were indeed surprised that neurogenesis was unaltered at E16.5 considering the basal position of the *Myh10* deficient progenitors. One possibility is this is due to the timing of the detachment (starting at E14.5) and our analysis of neurogenesis (2 days later at E16.5). It is possible that investigation of later stages of development would reveal altered neurogenesis. We have amended our discussion of *Myh9* and *Myh10* compensation in the cell body to take this into account (Lines 586-588). Regardless, we are confident that neurogenesis defects are not contributing to the detachment of basal endfeet from the pia between E14.5 and E16.5. Thus, importantly, this would not affect our overall conclusions regarding the role of endfeet in interneuron positioning. We also now include a discussion of possible roles for *Myh10* in neurogenesis (Lines 638-644).

Also, more representative images should be included for Fig 5J. Why is there hardly any staining seen for MYH10 at E15.5 while there is at E16.5?

To address the reviewer's concern about the representative image for Fig 5J (E15.5) we have replaced this with an image that shows MYH10 signal in the apical endfeet and adjacent cell bodies. We have also added an arrow to the figure and clarification in the figure legend to emphasize that the MYH10 signal of interest is at the ventricular border. We think this will help differentiate between the coronal sections in Fig 5J and en face view in Fig 5L.

4) The authors report that Myh9 and Myh10 transcripts localize to the basal endfeet with distinct temporal dynamics, with Myh9 mRNA being most enriched in the basal endfeet at E12.5 and declining at E13.5-E14.5 and Myh10 mRNA being low at early developmental stages (E12,5) and gradually increasing until the last stage examined (E16.5). Do the authors see similar results at the protein level? Of note, the authors should include more representative images for Fig. 3F, as the gradual increase of Myh10 mRNA over development is not very evident from the images shown.

Compared to *Myh9*, the change in *Myh10* mRNA localized to the endfeet is much more subtle. We have acknowledged this in the text referring to the change as a "gradual increase" (Line 242). It can be difficult to detect differences at the protein level by immunofluorescence; indeed *RNA in situ* is much more sensitive. As suggested, we have replaced the E16.5 image in Fig 3F. The new image shows the endfeet more tightly associated with each other and we believe this better exemplifies the increased smiFISH signal at this stage.

Also, the authors postulate that the Myh9 and Myh10 phenotypes may be driven by developmental timing when each isoform is enriched in the endfeet. They should further elaborate in the discussion how they envision the developmental timing could explain the divergent phenotypes observed for Myh9 and Myh10 cKO RGCs.

Perhaps NMHC II is required early in development to control RGC complexity and endfoot position, and later in development to regulate endfoot attachment to the BM. Therefore, due to their temporal localization patterns MYH9 may mediate these earlier functions whereas MYH10 mediates later functions. We have further elaborated on our discussion point of temporal regulation being a potential cause of divergent *Myh9* and *Myh10* phenotypes (Lines 580-583).

5) The authors present data supporting that Myh9 and Myh10 loss of function phenotypes are associated with altered distribution of interneurons in the marginal zone (MZ) and also increased interneuron number in the case of Myh10 loss. More representative images though should be included for Fig. 7G, and the authors should indicate that here the BM was labeled using anti-collagen I antibody. Perhaps beyond the scope of this study, it would be interesting to know the functional consequences of these alterations for cortical development and function.

The representative images for Fig. 7G were chosen as they most closely reflect the average value quantified for each condition. To clarify, the *Myh10* cKO brains do not show a reduction in the total number of LHX6+ interneurons at the basement membrane. Instead, we observe a reduction in the fraction of LHX6+ cells touching the BM relative to the total number of LHX6+ cells in the MZ. To clarify this, we have changed the Y-axis titles for the graphs in Fig. 7 F and H to read "LHX6+ cells at BM/ LHX6+ cells in MZ." Additionally, we reworded our description of these data in the results section to further clarify (Line 469-471).

We thank the reviewer for their careful reading of our figure legends. We have corrected the legend to state that COL1 was used to denote the BM in Fig. 7G rather than Laminin. We agree it will be valuable to understand functional consequences of interneuron distribution changes, a point we include in the discussion (Lines 634-635).

Reviewer #2: Radial glial cells perform many essential functions in the developing central nervous system. They are progenitors for neurons and glia, provide scaffolds for cell migration, give structural integrity to developing CNS structures, and help establish and maintain extracellular matrix structures like the basement membrane. Key to these functions of radial glial cells is their morphology. Radial glia form a bipolar pseudostratified epithelium, with their apical endfeet forming junctions at the ventricles and their basal endfeet forming attachments at the basement membrane between the brain and overlying meninges. These endfeet are important for several aspects of radial glia function, including their ability to attach to and remodel the basement membrane, maintain radial bipolar morphologies, and receive extracellular cues. However, most of the molecular mechanisms that establish radial glia endfeet and allow them to perform these functions are not known. An important barrier to progress in this area has been the small size of the endfeet compared to the rest of the developing brain, which limits access to study the molecules that are important for endfeet formation and function. In this manuscript, the authors develop a new approach for identifying proteins enriched in radial glia endfeet and test the functions of two such proteins in brain development.

Using biochemical, molecular, and genetic approaches in vivo, the authors make the following major, novel scientific findings: 1) Using in vivo proximity labeling in a novel way, they identify many proteins that are enriched in radial glia endfeet. 2) They validate several of these proteins by immunohistochemistry and some of their mRNAs by smiFISH, including non-muscle myosins MYH9 and MYH10 3) They show that conditional knockout of Myh9 in radial glia (and their progeny) causes defects in basal endfoot complexity and organization. 4) They find that conditional knockout of Myh10 causes a complete detachment of basal and apical endfeet. 5) They show that the endfeet defects caused by conditional knockout of Myh9 or Myh10 in radial glia cause non-cell-autonomous perturbations in interneuron organization. The authors conclude that these non-muscle myosins have distinct and complementary functions in radial glia endfeet organization and maintenance, and that proper endfeet function is required for normal organization of interneurons.

This study is relevant to readers of PLOS Biology who are interested in brain development, non-progenitor roles of radial glia, control and function of cell polarity and morphology, and cell migration and adhesion. The study is also of broader relevance and interest to those who study mechanisms of RNA/protein trafficking in highly polarized cells, as well as those interested in using in vivo approaches to uncovering the proteomes of subcellular structures. Overall, the experiments are well designed and executed. The data support the claims made, the presentation of the results is convincing, and the figures are easy to understand. The manuscript is well written and existing literature is appropriately cited. The methods are generally well-described. The study is outstanding for its creative use of in vivo biochemical approaches, state-of-the-art in vivo labeling and imaging of cells and subcellular structures, and solid genetic approaches.

In this reviewer's opinion, the following suggestions would improve the manuscript:

1. The "Control" genotype for genetic experiments is never defined in the results or methods. Should clarify the genotype(s) - fl/fl no cre, +/+ with cre, etc.

We have clarified in the methods and results (lines 272, 339-340, 673-674) that fl/fl no cre mice were used as controls for both *Myh9* and *Myh10* experiments.

2. Fig 6B-D - It is unclear how the MZ is defined. MZ thickness is typically assessed by the sparse nuclei situated between the pia and the densely populated top of layer 2. The image in 6B is cropped too much to tell if layer 2 is still obvious (more dense than the MZ) in the mutants? This should be clarified in the methods, along with how many measurements were taken (and presumably averaged) per brain.

MZ thickness was indeed assessed by the sparse nuclei between the pia and layer 2. Even with the additional cells in the *Myh10* cKO brains it is still possible to see a difference in density between the MZ and layer 2. Additionally, the orientation of the nuclei (more horizontal in the MZ and more vertical in layer 2) was used to validate the border in the densest areas. This information has been added to the newly created methods subsection “Measurement of MZ thickness and cells in MZ” (Lines 832-837). For measurements of cortical thickness, 3 measurements were averaged for each cortical section and 3 sections averaged per brain. This information has also been added to the methods section and clarified in the Fig 6 legend.

3. Fig 6C, H and Fig 7B, D - It should be clarified in the methods how cells in MZ were quantified. Presumably they were quantified per unit area, which should be reported (along with actual values, as in comment 4b below).

Cells in the MZ were identified as described above (density). We also measured the length of the BM in each image. The number of cells quantified in the MZ was normalized to 300µm of BM. This information has also been added to the “Measurement of MZ thickness and cells in MZ” subsection of the methods (Lines 832-837).

We have updated our graphs to report the data as actual values rather than normalized by litter. To represent litter variation, we have color-coded each litter and noted this in the figure legends.

4. Quantifications and graphs:
   a. Fig 4I and K - individual data points should be shown. In this case, a SuperPlot (see DOI 10.1083/jcb.202001064) would be more appropriate, in which the means for each brain (perhaps color-coded by litter) are plotted on top of the individual cells (color coded by brain). This will provide more information about possible brain to brain or litter to litter variability, beyond the cell to cell variability already reported in the current graph.

We thank the reviewers for suggesting SuperPlots. We were excited to use this method to represent both individual measurements and averages. We tried plotting these data (see below). Unfortunately, due to the discrete (non-continuous) nature of our data in Fig 4I and 4K, we do not believe this best represents our data. With this plot, the points representing the averages overlap with and hide each other. Additionally, the points representing single cells are crowded around the whole numbers. We could include these graphs in the supplement if the editors prefer.

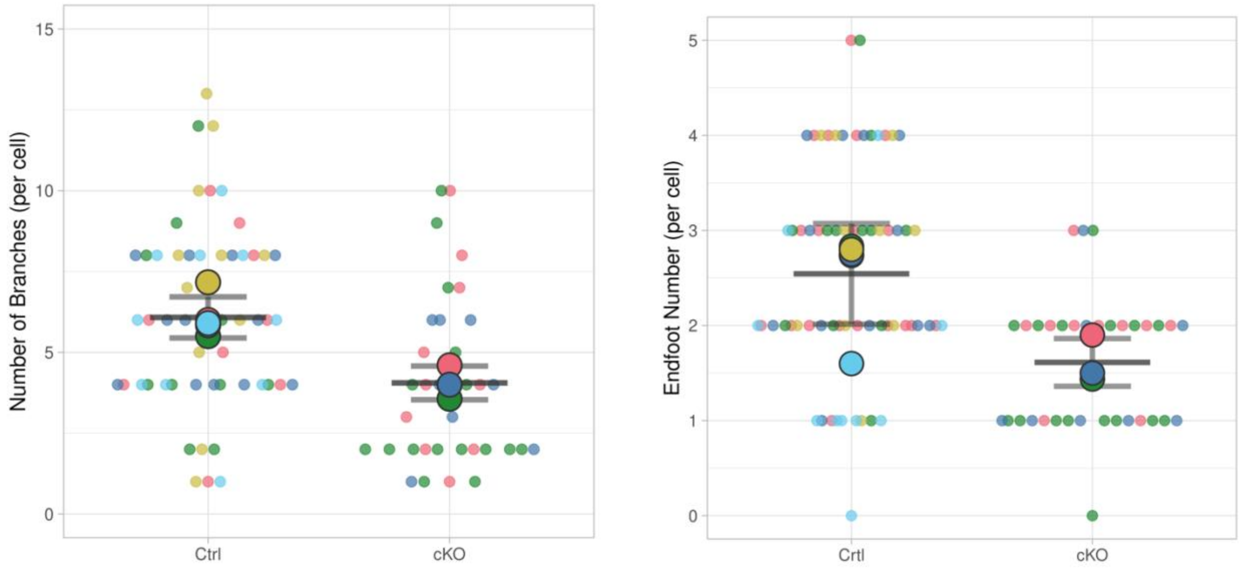

b. Fig 6H, 7B, and 7D - instead of presenting the data as "normalized by litter", consider presenting the actual values for each brain (n) in a SuperPlot. The data points for each brain can then be color coded (or otherwise represented, e.g., by different shapes) by litter, with averages of each litter superimposed (and possibly lines connecting littermates controls).

Due to the small number of brains quantified per litter (1-3) we do not feel a SuperPlot is the most informative way to represent these data. We updated our graphs to report the data as actual values rather than normalized by litter. To reflect litter variation, we have color-coded each litter and noted this in the figure legends.

5. The authors find more interneurons in the MZ of Myh10 mutants compared to Controls and conclude that more interneurons "flood into the open space of the MZ", possibly due to missing cues in the absence of radial glia endfeet. Although this is plausible, it would not be my first interpretation. Another possibility is that the INs that are migrating along their usual path in the MZ cannot subsequently migrate into the cortical plate properly. This could be because the radial glia scaffold and/or the general architecture of the cortical plate is perturbed. One simple way to assess this possibility would be to quantify the number of INs in the cortical plate and compare mutants to controls. If there are fewer INs in the cortical plate, this could mean that INs are getting stuck in the MZ (rather than "flooding" the MZ). If the number of INs in the cortical plate are not affected, it would be interesting to know where all the extra INs in the MZ came from. Is there a difference in their rostral-caudal or medial-lateral distribution? It would be informative and relatively straightforward to test these possibilities. At the very least they should be discussed as possible interpretations of the results.

We thank the reviewer for their insightful interpretation of these data. Due to the almost complete loss of endfeet and basal processes from the marginal zone by E16.5 in *Myh10* mutants, it is entirely possible that the interneurons do not have the scaffold they require to migrate into the cortical plate and thus, remain stranded in the MZ. We have added this important point to our discussion (Lines 625-627).

We have not observed any robust differences in interneuron distribution in the cortical plate or along rostral-caudal or medial-lateral axes. However, these are important questions that should

be carefully analyzed in future studies. We also discuss these points in the discussion (Lines 627-630).

6. Althought not the main point of the manuscript, the authors speculate that RGC basal endfeet may serve as hubs for local production and secretion of ECM components (lines 523-524). In this regard, it would be valuable to the readers if the authors discuss the fact that the basement membrane (staining for Laminin and COL1 in Fig 7) is not perturbed in the Myh9 and Myh10 knockouts. This is especially surprising for the Myh10 mutants that don't have endfeet at all. Perhaps the ECM production/secretion functions happen prior to the ages at which the endfeet detach?

We were also surprised by the relatively normal BM in the *Myh10* cKO mice given the lack of endfeet at the pia. We have not observed obvious defects with laminin or collagen staining. As suggested by the reviewer, we hypothesize that this could be due to the timing of the endfoot detachment. Perhaps endfeet are necessary for ECM secretion to build the BM early in development and are no longer vital for this role at E15.5 and E16.5 when the majority of endfeet are detached in *Myh10* mutants. Alternatively, it is possible that 2 days of endfoot detachment (E14.5-E16.5) is not sufficient to observe loss of the BM which may be visible later in development. We have added these points in our discussion (Lines 604-608).

Minor points:

1. Fig 3 - In the legend it is unclear why "n=" is reported since there are no quantifications. Does this mean that the images shown are representative of that many brains?

We have clarified in the figure legend that “n” is referring to the number of brains that were imaged in each condition to select the representative images shown.

2. Most instances of the word 'which' throughout the document should be changed to 'that'. Examples: lines 18, 89, 103, 108, etc (but not 99, 126, 153, etc).

We thank the reviewer for their detailed reading of our manuscript. We have fixed the word usage in these cases.

3. Line 153 - should clarify "endfoot proteins by IF and <mRNAs> by smiFISH"

We have added this clarification.

4. Lines 591-592 - An alternate interpretation is that the signal coordinating CR cell migration (CXCL12) is coming from the meninges, not the endfeet.
   a. Also relates to lines 597-598 when discussing interneuron migration. Since CR and IN migration are both regulated by CXCL12 from the pia (not endfeet), and CR position is unaffected, it seems unlikely that this signal is relevant to the Myh9 and Myh10 knockout phenotypes.

We agree with the reviewer that the CXCL12 regulating CR and IN migration is coming from the pia. Our intention was to use this as an example of an extracellular cue that is capable of regulating IN migration. We hypothesize that endfeet may also secrete additional extracellular cues that function in a similar way to further regulate IN migration. We have clarified this distinction in the text (lines 619-621).

Reviewer #3: This exciting manuscript convincingly demonstrates the first proteomic assessment of the end feet of RGCs and that MYH9 and MYH10 myosins have diverging functions in both endfeet branching complexity and end feet adhesion to the pial basement membrane.

While it is not surprising that MYH9 and MYH10 have diverging functions in this and other systems, the high quality of the work and the relevance neurological disorders where cellular interactions with neural cells with either the pial basement membrane and apical contacts in the VZ are common, and potential generalizability to other brain areas render this an outstanding contribution to our understanding of neural development.

With so many complex experiments in play, there are a fair number of issues that could be nit-picked, but this reviewer will refrain from doing so. This reviewer's opinion is that such suggestions would have minimal impact on the quality of the paper's message and would waste resources better left to the investigators for their next study. It would be interesting to compare and contrast adhesive mechanisms to the pial basement membrane and at apical attachments in the discussion section to more deeply explain some of the interesting phenotypes uncovered in the study.

We thank the reviewer for their thoughtful points and enthusiasm for our study. We have added these suggestions regarding adhesive mechanisms to the discussion (Lines 644-648).
